# Apigenin, a non-mutagenic dietary flavonoid, suppresses lupus by inhibiting autoantigen presentation for expansion of autoreactive Th1 and Th17 cells

**DOI:** 10.1186/ar2682

**Published:** 2009-04-30

**Authors:** Hee-Kap Kang, Diane Ecklund, Michael Liu, Syamal K Datta

**Affiliations:** 1 Division of Rheumatology, Departments of Medicine and Microbiology-Immunology, Northwestern University Feinberg School of Medicine, 240 East Huron Street, Chicago, IL 60611, USA

## Abstract

**Introduction:**

Lupus patients need alternatives to steroids and cytotoxic drugs. We recently found that apigenin, a non-mutagenic dietary flavonoid, can sensitize recurrently activated, normal human T cells to apoptosis by inhibiting nuclear factor-kappa-B (NF-κB)-regulated Bcl-x_L_, cyclooxygenase 2 (COX-2), and cellular FLICE-like inhibitory protein (c-FLIP) expression. Because sustained immune activation and hyperexpression of COX-2 and c-FLIP contribute to lupus, we treated SNF1 mice that spontaneously develop human lupus-like disease with apigenin.

**Methods:**

SNF1 mice with established lupus-like disease were injected with 20 mg/kg of apigenin daily and then monitored for development of severe nephritis. Histopathologic changes in kidneys, IgG autoantibodies to nuclear autoantigens in serum and in cultures of splenocytes, along with nucleosome-specific T helper 1 (Th1) and Th17 responses, COX-2 expression, and apoptosis of lupus immune cells were analyzed after apigenin treatment.

**Results:**

Apigenin in culture suppressed responses of Th1 and Th17 cells to major lupus autoantigen (nucleosomes) up to 98% and 92%, respectively, and inhibited the ability of lupus B cells to produce IgG class-switched anti-nuclear autoantibodies helped by these Th cells in presence of nucleosomes by up to 82%. Apigenin therapy of SNF1 mice with established lupus suppressed serum levels of pathogenic autoantibodies to nuclear antigens up to 97% and markedly delayed development of severe glomerulonephritis. Apigenin downregulated COX-2 expression in lupus T cells, B cells, and antigen-presenting cells (APCs) and caused their apoptosis. Autoantigen presentation and Th17-inducing cytokine production by dendritic cells were more sensitive to the inhibitory effect of apigenin in culture, as evident at 0.3 to 3 μM, compared with concentrations (10 to 100 μM) required for inducing apoptosis.

**Conclusions:**

Apigenin inhibits autoantigen-presenting and stimulatory functions of APCs necessary for the activation and expansion of autoreactive Th1 and Th17 cells and B cells in lupus. Apigenin also causes apoptosis of hyperactive lupus APCs and T and B cells, probably by inhibiting expression of NF-κB-regulated anti-apoptotic molecules, especially COX-2 and c-FLIP, which are persistently hyperexpressed by lupus immune cells. Increasing the bioavailability of dietary plant-derived COX-2 and NF-κB inhibitors, such as apigenin, could be valuable for suppressing inflammation in lupus and other Th17-mediated diseases like rheumatoid arthritis, Crohn disease, and psoriasis and in prevention of inflammation-based tumors overexpressing COX-2 (colon, breast).

## Introduction

In lupus, intrinsic 'hyperactivity' of the immune system is associated with persistent interactions between certain autoimmune T helper (Th) cells and B cells, leading to the production of IgG autoantibodies against apoptotic nuclear antigens and the formation of pathogenic immune complexes [1,2]. Normally, autoreactive T and B cells are eliminated by functional inactivation (anergy) and activation-induced cell death (AICD) (apoptosis) [3]. However, autoimmune Th cells of human lupus resist AICD by upregulating the expression of cyclooxygenase 2 (COX-2) and the anti-apoptotic molecule c-FLIP (cellular FLICE-like inhibitory protein) in a sustained manner [4]. COX-2 is also overexpressed and is important for survival and function of other cells involved in the autoimmune inflammatory responses for pathogenesis of lupus [5,6]. Therefore, COX-2 and associated molecules are critical targets for developing non-mutagenic steroid-sparing drugs for lupus therapy. Indeed, intermittent therapy with low doses of the COX-2 inhibitor celecoxib (Celebrex) has beneficial effects in murine models of lupus [6,7], and preliminary results are encouraging in lupus patients [8].

Apigenin (4',5,7-trihydroxyflavone) is a non-toxic non-mutagenic flavonoid that is widely distributed in dietary plants, especially in parsley, thyme, peppermint, olives, and herbs like chamomile, and it can block COX-2 expression in cancer cells [9]. We found that, in chronically activated but not in freshly activated human T cells, relatively non-toxic apigenin can suppress PI3K-Akt-mediated nuclear factor-kappa-B (NF-κB) activation and, consequently, NF-κB-regulated anti-apoptotic pathways, especially inhibiting c-FLIP and COX-2 expression that are important for functioning and maintenance of immune cells in inflammation, autoimmunity, and lymphoproliferation [5]. Although apigenin decreases COX-2 expression, it does not counteract COX-2 enzymatic activity itself. Moreover, unlike the conventional COX-2 inhibitors, celecoxib (Celebrex), rofecoxib (Vioxx), or other non-steroidal anti-inflammatory drugs, apigenin has vasorelaxing, anti-platelet, and anti-oxidant properties, which could reduce the risk of coronary disease and improve endothelial function [10-13]. Herein, we treated spontaneously developing systemic lupus erythematosus in the (SWR × NZB)F1 (SNF1) mouse model [14,15] with apigenin and studied its mechanistic effects on the lupus immune system.

## Materials and methods

### Mice

NZB and SWR mice were purchased from The Jackson Laboratory (Bar Harbor, ME, USA). Lupus-prone SNF1 hybrids were bred and females were used with the approval of the Animal Care and Use Committee (ACUC).

### Administration of apigenin

Apigenin was purchased from Sigma-Aldrich (St. Louis, MO, USA) and dissolved in dimethyl sulfoxide (DMSO) and then diluted in phosphate-buffered saline (PBS) for experiments. Twelve-week-old SNF1 mice were injected intraperitoneally with apigenin (3, 6, or 20 mg/kg) daily. The control group was injected with the same amount of vehicle solution (DMSO-PBS). All mice were monitored weekly for the development of proteinurea by testing with Albustix (VWR International, West Chester, PA, USA) and for survival. The treatment lasted until the mice were 52 weeks old. To study early immunologic changes after treatment with apigenin, additional batches of 12-week-old SNF1 mice (five mice per group) were treated with the same regimens as described above and then sacrificed after 8 weeks.

### Quantitation of total IgG and IgG autoantibodies

IgG class autoantibodies to single-stranded DNA (ssDNA), double-stranded DNA (dsDNA), histone, and nucleosome (histone-DNA complex) were measured by enzyme-linked immunosorbent assay (ELISA) [16,17]. Two months after apigenin treatment (at 5 months of age), the SNF1 mice were bled for autoantibody measurement in serum. Total IgG and IgG subclasses in sera of apigenin- or vehicle-treated SNF1 mice were also quantitated by ELISA [16-18]. Briefly, 96-well plates were coated with goat anti-mouse IgG antibody (SouthernBiotech, Birmingham, AL, USA). Serially diluted serum samples were added and incubated overnight and then total IgG or IgG subclasses were measured by using goat anti-mouse IgG-alkaline phosphatase (AP) conjugate or anti-mouse IgG isotype-specific antibody-AP conjugates.

### Measurement of intracellular cyclooxygenase 2 and analysis surface marker staining by flow cytometry

Three-month-old SNF1 female mice were treated with apigenin (20 mg/kg) or vehicle solution for 8 weeks. Total spleen cells from apigenin- or vehicle-treated SNF1 mice were stained with fluorescein isothiocyanate-conjugated antibodies to mouse CD4 (for T cells), mouse CD19 and CD86 for activated B cells, mouse CD11c for dendritic cells (DCs), and mouse F4/80 for macrophages (BD Pharmingen, San Diego, CA, USA, or eBioscience, San Diego, CA, USA, respectively) at 4°C for 30 minutes. Antibody to CD11b was also used, but it is a marker shared by DC subsets, macrophages, and other cell types. After washing and fixation, cells were permeabilized and stained with goat anti-mouse COX-2 antibody or its isotype control conjugated with phycoerythrin (Santa Cruz Biotechnology, Inc., Santa Cruz, CA, USA) at room temperature for 30 minutes. To lower the background for intracellular staining, we used cell fixation and permeabilization reagents from eBioscience (00-5523) and a different antibody for COX-2 staining (sc-1745; Santa Cruz Biotechnology, Inc.) than in a previous study [6]. For analysis, isotype-matched control stainings were used for marking positive and negative cell populations. Usually, 200,000 events were collected after live cell gating, using FACSCalibur, and analyzed by CellQuest (BD Pharmingen) or FlowJo software (TreeStar Inc., FlowJo LLC, Ashland, OR, USA).

### Induction of apoptosis

Spleen cells from 6-month-old SNF1 mice were cocultured for 24 hours with various concentrations of apigenin to measure experimental apoptosis or vehicle to measure spontaneous, control apoptosis. Apoptotic cells were detected by staining of whole splenocytes with annexin V and propidium iodide (BD Pharmingen), accompanied by simultaneous staining with appropriate fluorochrome-conjugated antibodies to CD4, B220, CD19, CD11c, or CD11b. Apoptosis in the specific cell subset, as gated by flow cytometry, was calculated as (percentage of experimental apoptosis – percentage of spontaneous apoptosis)/(100 – percentage of spontaneous apoptosis) [4].

### Enzyme-linked immunosorbent spot assay

Enzyme-linked immunosorbent spot (ELISPOT) assay plates (Cellular Technology Ltd., Shaker Heights, OH, USA) were coated with capture antibodies against interferon-gamma (IFN-γ) (BD Pharmingen) in PBS at 4°C overnight. Splenic T cells (1 × 10^6^) from treated mice were cultured with irradiated (3,000 rad) splenic antigen-presenting cells (APCs) (non-T cells) from 1-month-old SNF1 mice in the presence of nucleosomes or their peptide autoepitopes or of PBS control. Cells were removed after 24 hours of incubation for IFN-γ or after 48 hours for interleukin (IL)-17 production, and the responses were visualized by the addition of the individual anti-cytokine antibody-biotin and subsequent horseradish peroxidase-conjugated streptavidin. Cytokine-expressing cells were detected by Immunospot scanning and analysis (Cellular Technology Ltd.). To test the effect of apigenin on nuclear autoantigen presentation, apigenin- or vehicle-pulsed splenic T cells (5 × 10^5 ^per well) were cocultured with apigenin- or vehicle-pulsed APCs (5 × 10^5 ^per well) for 1 hour before being added to IFN-γ or IL-17 ELISPOT plates. The cultures were performed in the presence of 0.1 to 30 μg/mL nucleosomes.

### Cytokine enzyme-linked immunosorbent assay

DCs (5 × 10^5^) from apigenin- or vehicle-treated SNF1 mice were isolated as described [18] and stimulated with nucleosomes (1 to 60 μg/mL) or Toll-like receptor (TLR)-9 ligand CpG or TLR-7 ligand R837 (1 to 100 μg/mL) obtained from InvivoGen (San Diego, CA, USA). After 60 hours, amounts of IL-6 in culture supernatants were measured by BD OptEIA™ ELISA set (BD Pharmingen).

### Helper assays for IgG autoantibody production

To test the effect of apigenin on IgG autoantibody production *in vitro*, whole splenocytes (1 × 10^6 ^cells per well) were stimulated with 10 μg/mL nucleosomes in the presence of various amounts of apigenin or vehicle solution. After 7 days of culture, supernatants were collected and assayed by ELISA for IgG antibodies against dsDNA, ssDNA, histones, and nucleosomes as described [17].

### Histopathologic analysis of kidneys

Halves of each kidney from apigenin- or control vehicle-treated mice were fixed in 10% formalin and paraffin-embedded. To determine the extent of renal disease, sections were stained with hematoxylin and eosin and periododic acid-Schiff and graded in a blinded fashion from 0 to 4+ for pathologic changes (as described in [17,19-21]).

### Statistical analysis

The log-rank test and the Student two-tailed *t *test were used. Results are expressed as mean ± standard error of the mean unless noted otherwise.

## Results

### Apigenin suppresses interferon-gamma response to nuclear autoantigen and IgG autoantibody production *in vitro*

**Figure 1:**
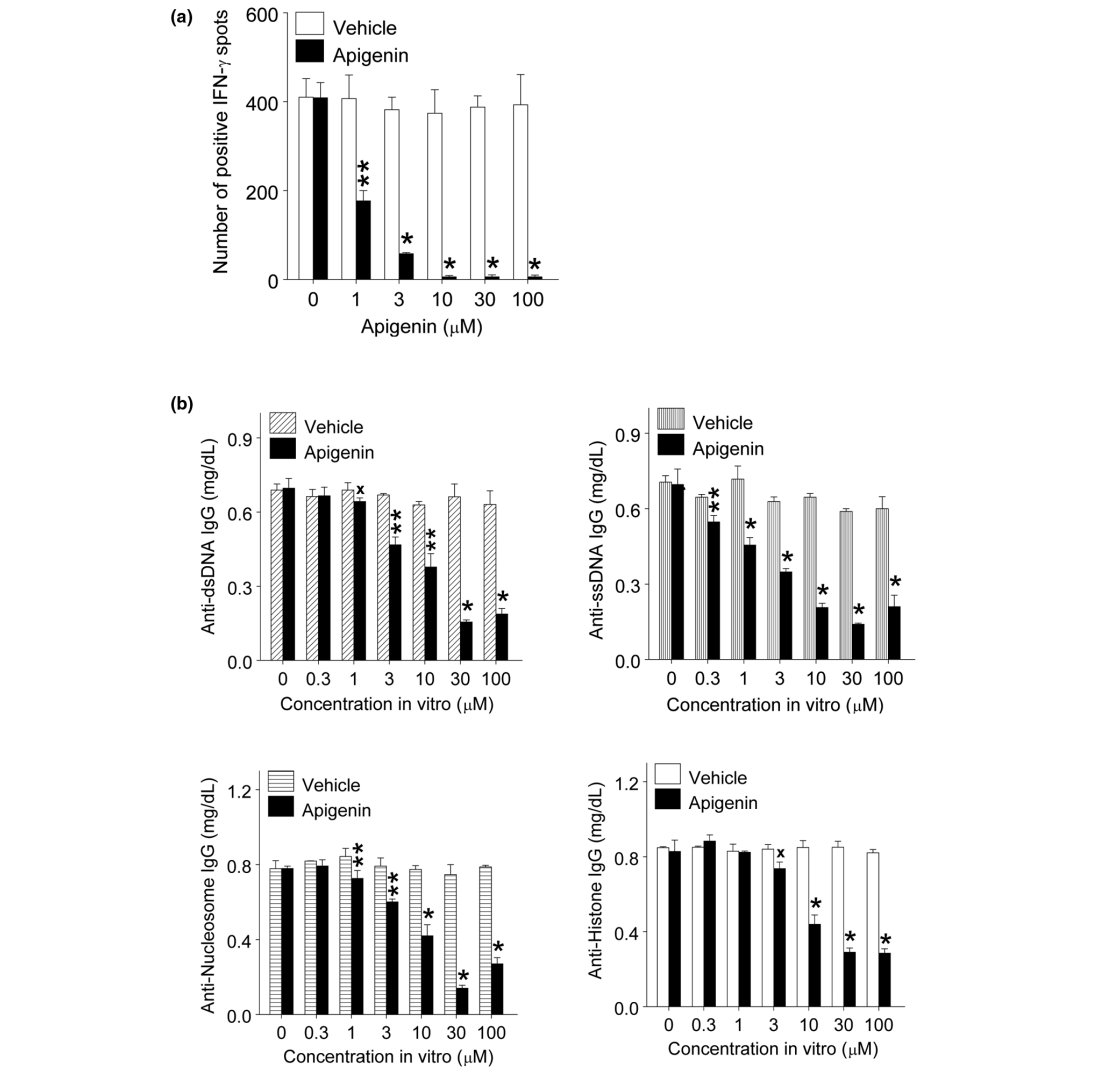
Apigenin suppressed nucleosome-specific interferon-gamma (IFN-γ) response and IgG-autoantibody production. Splenocytes from 5- to 6-month-old unmanipulated SNF1 mice were stimulated with nucleosomes in the presence of various amounts of apigenin or vehicle (dimethyl sulfoxide-phosphate-buffered saline). **(a) **Apigenin markedly suppressed IFN-γ responses by nucleosome-specific T cells in enzyme-linked immunosorbent spot assay. **(b) **Apigenin significantly reduced the level of IgG class autoantibodies in nucleosome-stimulated lupus Th cell-B cell coculture assays. *P < 0.001, **P < 0.01, and ^x^*P *< 0.02. dsDNA, double-stranded DNA; SNF1, (SWR × NZB)F1; ssDNA, single-stranded DNA.

T cells in unmanipulated SNF1 mice are spontaneously primed to nuclear autoantigens in early life and respond to them *ex vivo *by proliferation and production of IFN-γ without further immunization [17,22]. Splenocytes from 5- to 6-month-old SNF1 mice with overt lupus renal disease were stimulated *in vitro *with nucleosomes (3 μg/mL) in the presence of various amounts of apigenin (1 to 100 μM) and then analyzed for IFN-γ production by ELISPOT. IFN-γ responses to nucleosomes were markedly reduced by apigenin as compared with vehicle (Figure 1a, *P *< 0.01 to 0.001). Exposure to 1 μM apigenin reduced the response to autoantigen by 57%, 3 μM apigenin inhibited response by 85%, and apigenin at concentrations of 10 μM or above reduced the autoimmune response by 98% (Figure 1a).

We also found that the levels of IgG class anti-dsDNA, anti-ssDNA, anti-nucleosome, and anti-histone autoantibodies in culture supernantants of nucleosome-stimulated SNF1 mouse splenocytes were significantly reduced (up to 77%, 76%, 82%, and 66%, respectively) in the presence of apigenin (0.3 to 100 μM) in comparison with vehicle (Figure 1b, *P *< 0.05 to 0.001). In this helper assay, the splenocytes were cultured for 7 days, and apigenin or vehicle was added once at the beginning of culture. Thus, significant suppression of IFN-γ responses to nucleosomes and reduction of IgG class-switched autoantibody production occurred with 0.3 to 100 μM apigenin (Figure 1).

### Optimal dose of apigenin *in vivo *for suppression of interferon-gamma response to nucleosomes

**Figure 2:**
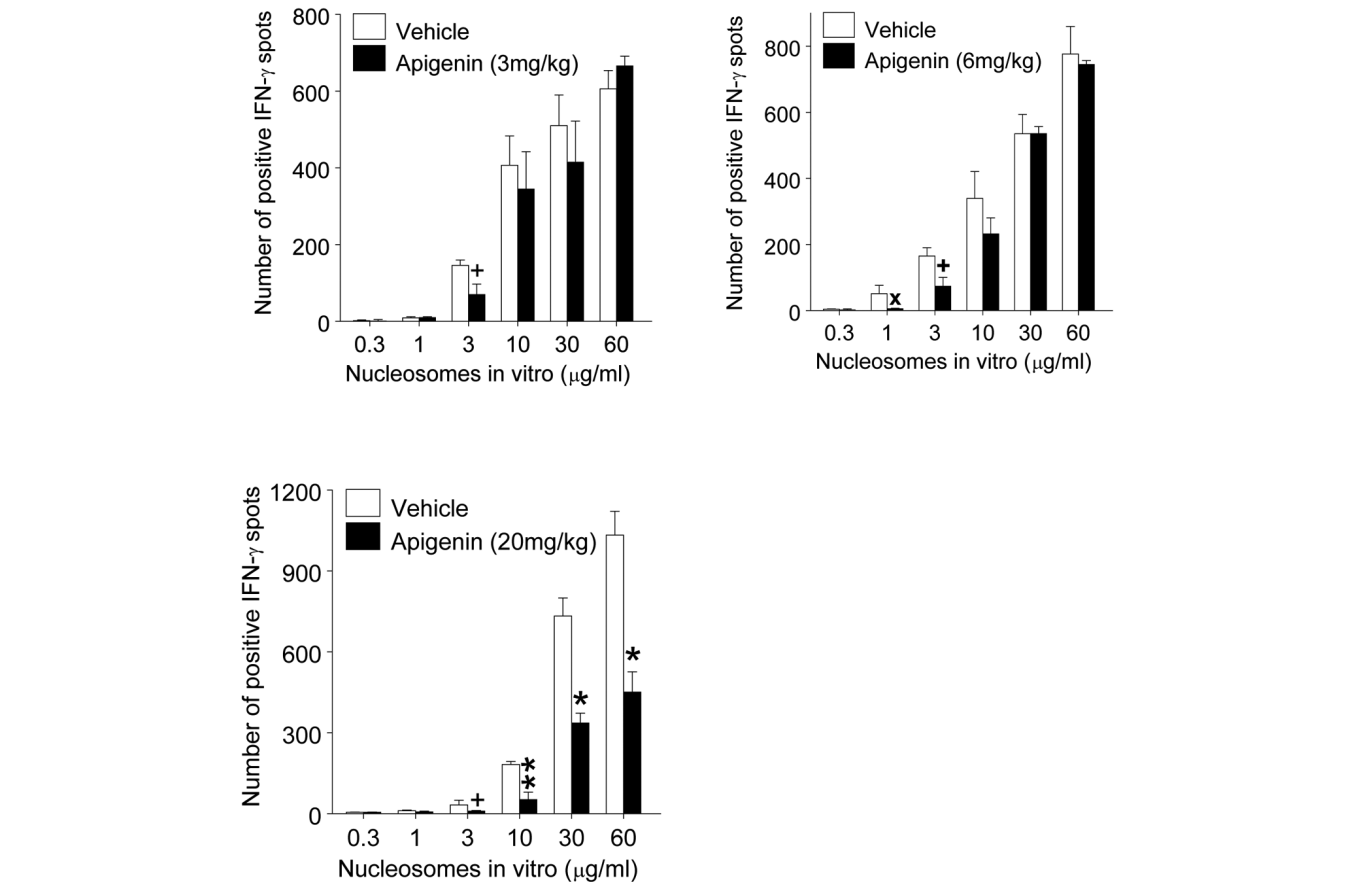
Dose response for *in vivo *treatment with apigenin for suppressing interferon-gamma (IFN-γ) response to nucleosomes. Three-month-old unmanipulated SNF1 mice were treated daily with apigenin at 3 mg/kg (13.89 μM), 6 mg/kg (27.8 μM), and 20 mg/kg (0.93 mM). Treatment with 20 mg/kg apigenin for 2 weeks markedly suppressed IFN-γ response to nuclesosomes *ex vivo*. Values are mean ± standard error of the mean. *P < 0.001, **P < 0.01, ^x^*P *< 0.02, and ^+^*P *< 0.05. SNF1, (SWR × NZB)F1.

We used the suppressive effect of apigenin on lupus spleen cells' IFN-γ response to nucleosomes (Figure 1) to determine the optimal dose for *in vivo *treatment. We injected unmanipulated 3-month-old SNF1 mice intraperitoneally with apigenin daily at 3 mg/kg (13.89 μM), 6 mg/kg (27.8 μM), and 20 mg/kg (0.93 mM). At this age, the SNF1 mice have elevated levels of anti-nuclear autoantibodies in serum, but they do not have overt proteinuria. After 2 weeks of treatment, we tested splenocytes from treated mice for IFN-γ response to various amounts of nucleosomes *ex vivo*. Although injection treatment with the lowest dose had an inhibitory effect on IFN-γ response to the autoantigen *ex vivo*, the 20 mg/kg dose showed the most marked suppression in responses even at higher doses of the autoantigen (Figure 2, *P *< 0.05 to 0.001). Therefore, we decided to use a concentration of 20 mg/kg (0.93 mM) for *in vivo *treatment. Moreover, apigenin administration was found to be non-toxic at 20 mg/kg in other situations [23].

### *In vivo *treatment with apigenin suppresses interferon-gamma and interleukin-17 responses and IgG autoantibody production to nucleosomes

**Figure 3:**
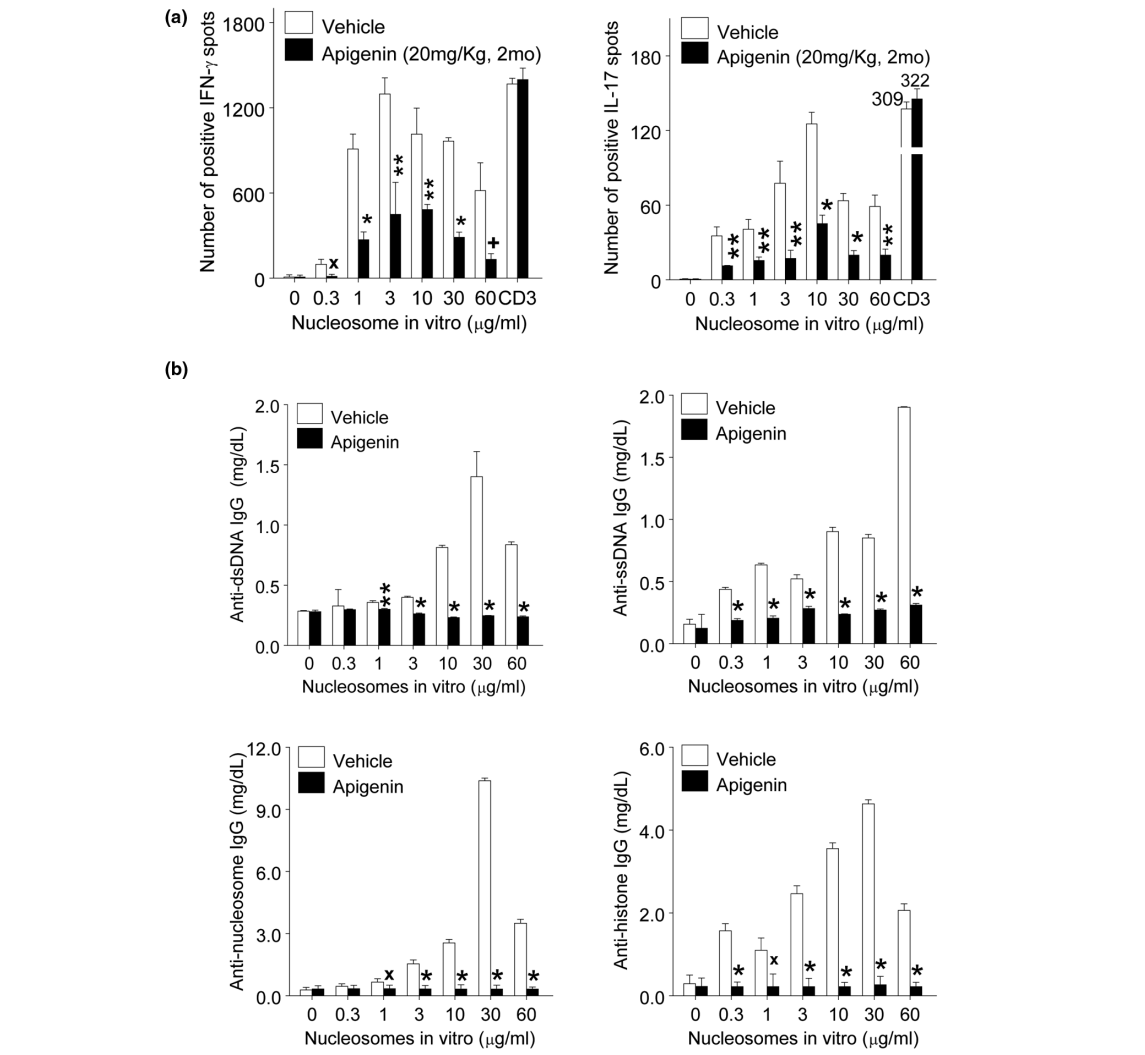
*In vivo *treatment with apigenin reduced nucleosome-specific Th1, Th17, and IgG autoantibody production. *In vivo *treatment with apigenin (20 mg/kg) for 2 months markedly reduced nucleosome-specific Th1 and Th17 responses and IgG autoantibody production *ex vivo *as compared with vehicle-treated SNF1 mice. **(a) **Splenocytes from apigenin- or vehicle-treated SNF1 mice were stimulated with nucleosomes and analyzed for Th1(left panel) and Th17 (right panel) responses by enzyme-linked immunosorbent spot assay. 'CD3' indicates results upon stimulation with optimal amount of anti-CD3 antibody (0.2 μg/mL). **(b) **IgG autoantibody levels of anti-dsDNA, anti-ssDNA, anti-nucleosome, and anti-histone in culture supernatants of lupus Th cell-B cell-nucleosome cocultures were analyzed by enzyme-linked immunosorbent assay. *P < 0.001, **P < 0.01, ^x^*P *< 0.02, and ^+^*P *< 0.05. dsDNA, double-stranded DNA; IL-17, interleukin-17; SNF1, (SWR × NZB)F1; ssDNA, single-stranded DNA; Th, T helper.

Three-month-old SNF1 mice were treated by intraperitoneal injection with 20 mg/kg apigenin or vehicle daily. After 2 months of treatment, we analyzed IFN-γ and IL-17 responses of nucleosome-specific T cells and IgG autoantibody responses by culturing splenocytes from apigenin- or vehicle-treated SNF1 in the presence of various concentrations of nucleosomes. We found that IFN-γ and IL-17 responses to nucleosome by lupus T cells were markedly reduced as compared with vehicle treatment (up to 79% and 88%, respectively) (Figure 3a, *P *< 0.05 to 0.001). However, polyclonal Th1 and Th17 responses with low-dose or optimal anti-CD3 (0.2 μg/mL) stimulation were not suppressed by apigenin treatment (Figure 3a). Moreover, we did not observe any significant differences in viability of spleen cells isolated from apigenin-treated and vehicle-treated mice. We also observed significant reductions (up to 83%, 84%, 97%, and 94%, respectively) in the levels of IgG class anti-dsDNA, anti-ssDNA, anti-nucleosomes, and anti-histone autoantibodies in culture supernantants of nucleosome-stimulated splenocytes from apigenin-treated SNF1 mice as compared with vehicle-treated mice (Figure 3b, *P *< 0.02 to 0.001).

### Apigenin therapy suppresses IgG autoantibody levels in serum and delays incidence of severe renal disease

**Figure 4:**
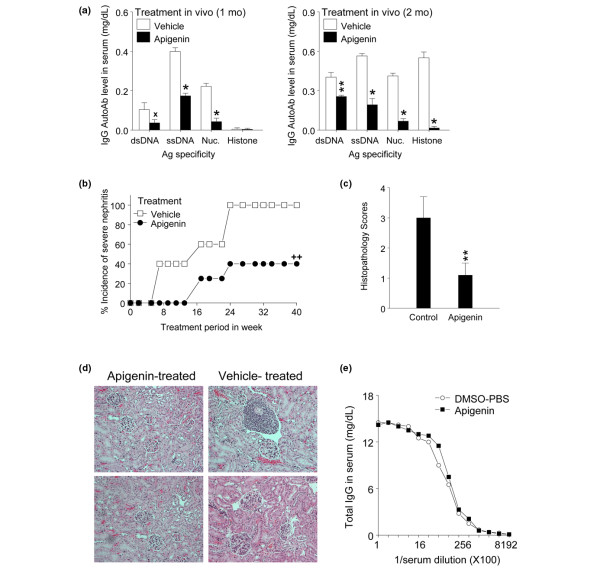
Apigenin treatment *in vivo *suppresses IgG anti-nuclear autoantibodies and lupus nephritis. **(a) **Treatment for 1 month and 2 months resulted in significant reduction of IgG autoantibody levels in serum of SNF1 mice as compared with vehicle treatment. **(b) **Another group of mice was treated with apigenin or vehicle and monitored for the incidence of severe nephritis. Apigenin treatment markedly delayed incidence of nephritis (log-rank test, ^++^*P *= 0.00313). **(c) **With treatment regimens identical to those in (b), renal histopathologic features of lupus nephritis were evaluated. Apigenin treatment significantly lowered the histopathology score of nephritis. **(d) **Representative histopathology figures of kidneys with treatment regimens identical to those in (b); hematoxylin and eosin stain (× 200). **(e) **Total IgG levels in serum of apigenin- or vehicle-treated mice were measured by enzyme-linked immunosorbent assay. *P < 0.001, **P < 0.01, and ^x^*P *< 0.02. Ag, antigen; AutoAb, autoantibody; DMSO-PBS, dimethyl sulfoxide-phosphate-buffered saline; dsDNA, double-stranded DNA; Nuc, nucleosome; SNF1, (SWR × NZB)F1; ssDNA, single-stranded DNA.

We injected apigenin (20 mg/kg) into 3-month-old unmanipulated SNF1 mice intraperitoneally. After 1 month and 2 months of treatment with daily intraperitoneal injections of apigenin, we measured IgG autoantibody levels in serum by ELISA. Treatment for 1 month reduced IgG class autoantibodies to dsDNA, ssDNA, and nucleosomes by 65%, 57%, and 81%, respectively (Figure 4a, *P *< 0.02, *P *< 0.001, and *P *< 0.001, respectively), and after 2 months of treatment, the levels of the respective IgG autoantibodies were reduced by 37%, 66%, 83%, and 97% (Figure 4b, *P *< 0.01, *P *< 0.001, *P *< 0.001, and *P *< 0.001, respectively). However, apigenin treatment did not result in reduction of total IgG levels in serum (Figure 4e), and the distribution of total IgG isotypes was not changed by apigenin treatment as compared with vehicle-treated control mice (data not shown).

Another batch of 3-month-old pre-nephritic SNF1 mice (10 mice per group) were injected intraperitoneally daily with apigenin (20 mg/kg) or DMSO-PBS vehicle as control. The control group started developing severe nephritis from 20 weeks of age, as documented by persistent proteinurea of greater than 100 mg/dL (Figure 4b, log-rank test, *P *= 0.00313) and a renal pathology grade of 3 to 4+ (Figure 4c, *P *< 0.01). From 18 to 24 weeks of age, 40% of control group mice developed severe nephritis, whereas apigenin-injected mice did not develop overt renal disease. At 36 weeks of age, 100% of control group mice had developed severe nephritis, whereas only 40% of apigenin-injected group developed severe nephritis.

At 42 to 52 weeks of age, 20% of DMSO-PBS-treated mice were dead, whereas 100% of apigenin-treated mice were alive. However, survival curves of mice followed until death cannot be shown as moribund mice with severe nephritis had to be euthanized according to ACUC rules. There were no gross signs of toxicity or apparent loss of weight in the apigenin-treated mice as compared with age-matched normal strains, such as SWR or C57B/L6 mice, consistent with other studies [23]. Weight gain, apparently due to fluid retention and lethargy, was observed in mice of either group after they had developed severe nephritis and proteinuria.

For assessment of renal pathologic features at the earliest stages (before persistent proteinuria sets in), another group of 3-month-old mice was treated for 6 weeks. Kidney sections from control and apigenin-treated mice were examined and graded for typical lesions of lupus glomerulonephritis such as glomerular enlargement, hypercellularity, crescent formation, mesangial thickening, glomerulosclerosis, and interstitial infiltration with mononuclear cells [17,19-21]. Six weeks after apigenin treatment, kidney sections from control mice had an overall score of 3 ± 0.7 for nephritis, whereas the apigenin-treated group showed 1.1 ± 0.4 as the overall score (Figures 4c and 4d, *P *< 0.001).

**Figure 5:**
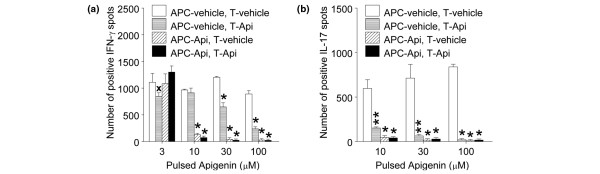
Effect of apigenin on nucleosome-induced Th1 and Th17 responses and antigen presentation function of antigen-presenting cells (APCs). T cells and APCs from 3-month-old unmanipulated SNF1 mice were pulsed with various amounts of apigenin or vehicle for 1 hour, and crisscross cocultures were done in the presence of nucleososome (10 μg/mL). Apigenin pre-exposure suppressed autoantigen-presenting ability of APCs and resulted in inhibition of Th1 **(a) **and Th17 **(b) **responses more markedly than pre-exposure of the responding T cells to apigenin. *P < 0.001, **P < 0.01, and ^x^*P *< 0.02. Api, apigenin; IFN-γ, interferon-gamma; IL-17, interleukin-17; SNF1, (SWR × NZB)F1; Th, T helper.

Antigen-presenting cells are more sensitive to apigenin than T cells in suppression of nucleosome-specific interferon-gamma and interleukin-17 responsesWe tested which cells are sensitive to apigenin in suppression of autoantigen response. We pulsed APCs and T cells isolated from splenocytes from 4- to 5-month-old SNF1 mice with apigenin or vehicle for 1 hour and then cultured apigenin-treated APCs with vehicle-treated T cells, and apigenin-treated T cells with vehicle-treated APCs in the presence of various amounts of nucleosomes, and then analyzed for IFN-γ and IL-17 ELISPOT responses. APCs were more sensitive to apigenin than T cells. Apigenin-pulsed APCs showed marked reduction of nucleosome-specific IFN-γ response at 10 to 100 μM, whereas apigenin-pulsed T cells showed marked reduction in IFN-γ response at 30 to 100 μM. In the case of nucleosome-specific IL-17 response, both apigenin-pulsed APCs and T cells showed marked reduction at 10 to 100 μM, but apigenin-pulsed APCs showed more reduction than T cells (Figures 5a and 5b, *P *< 0.02 to 0.001). At a concentration of 10 μM, apigenin pre-treated APCs showed 87% reduction of autoimmune IFN-γ response as compared with that of vehicle-treated APCs, whereas apigenin pre-treated T cells showed only 6% reduction, and at the same concentration, apigenin pre-treated APCs showed 92% reduction of autoimmune IL-17 responses, whereas apigenin pre-treated T cells showed 75% reduction.

### Apigenin treatment reduces the level of cyclooxygenase 2 in lupus CD4^+ ^T cells, B cells, dendritic cells, and macrophages

**Figure 6:**
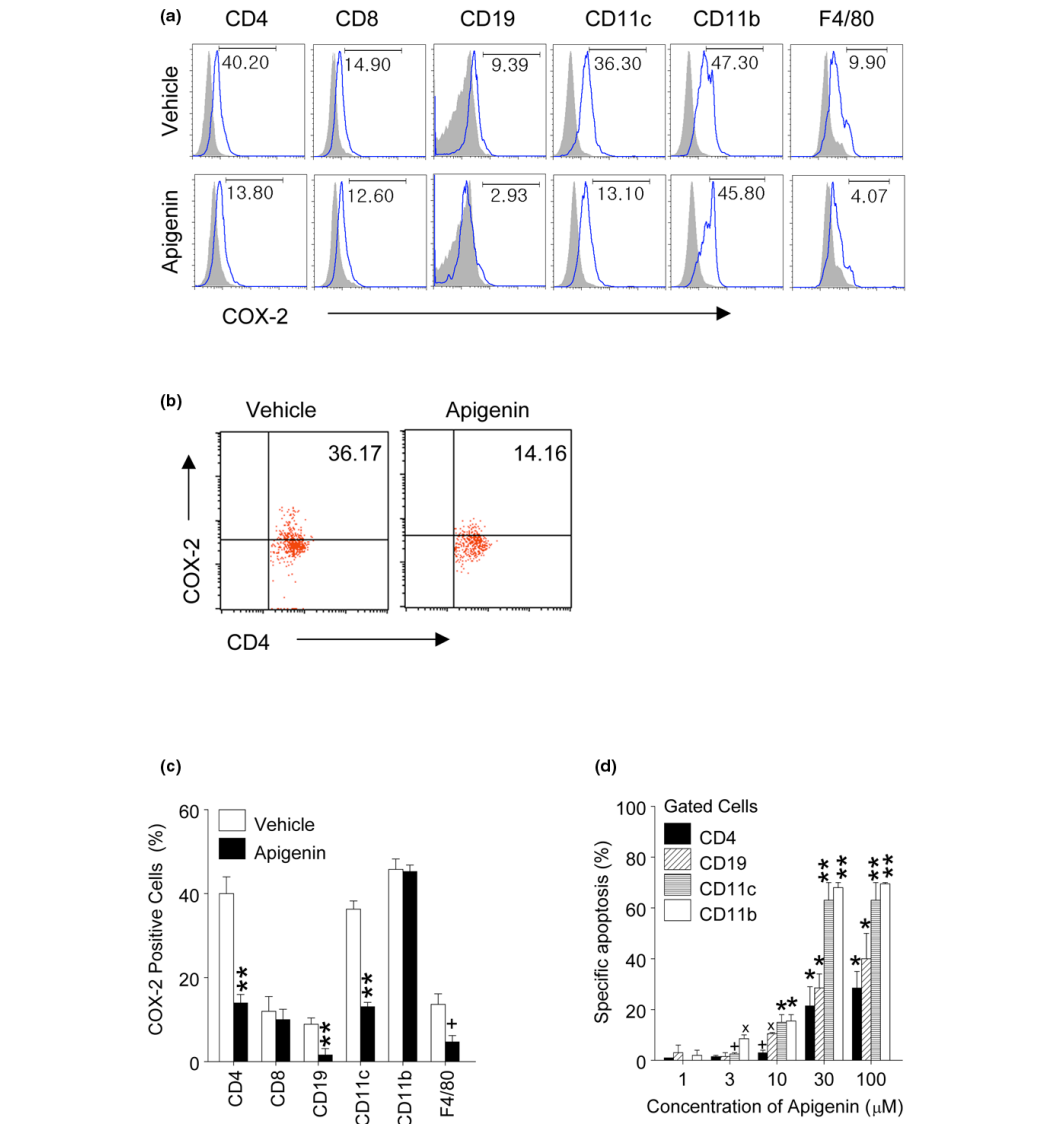
Effect of apigenin on cyclooxygenase 2 (COX-2) expression and apoptosis. Intracellular COX-2 expression followed treatment with apigenin or vehicle for 3 months. **(a) **COX-2 expression in representative histograms of spleen cell subsets. **(b) **Representative dot plot of gated CD4 T cells (percentage shown in right upper quadrant). **(c) **Compiled results from three experiments. Treatment with apigenin markedly suppressed COX-2 expression in gated CD4^+ ^T cells, B cells, dendritic cells (DCs), and macrophages, but there was no difference in total CD11b^+ ^cells or CD8^+ ^T cells. **(d) ***In vitro *treatment with apigenin induced apoptosis of lupus T cells, B cells, DCs, and macrophages from SNF1 mice after 24-hour incubation. Culture with 30 μM apigenin resulted in a twofold increase in percentage of specific apoptosis in DCs and macrophages than in T and B cells. Apoptotic cells were analyzed in gated cell subsets to calculate percentage of specific apoptosis, as described in Materials and methods (n = 5 per stain). *P < 0.001, **P < 0.01, ^x^*P *< 0.02, and ^+^*P *< 0.05 for (c) and (d). SNF1, (SWR × NZB)F1.

Since SNF1 mouse T cells, activated B cells, DCs, and macrophages express higher basal levels of COX-2 as compared with those in non-autoimmune SWR or BALB/c strains and hyperexpression of COX-2 contributes to lupus autoimmunity [4,6], we tested whether apigenin could reduce hyperexpression of COX-2 in cells of autoimmune SNF1 mice. After 3 months of treatment with apigenin (20 mg/kg daily), COX-2 expression was markedly reduced in CD4^+ ^T cells, B cells, DCs, and macrophages (but there were no differences in total CD11b^+ ^cells or CD8^+ ^cells) (Figures 6a and 6c, *P *< 0.05 to 0.01). A high proportion of activated lupus cells (particularly CD4 T cells and DCs) expressed COX-2 (Figure 6c), and it appeared that apigenin caused depletion of these COX-2-positive cells. However, apigenin treatment resulted in the apparent removal of only the cells expressing high levels of COX-2 (Figure 6b). CD4^+ ^T cells in apigenin-treated mice were still expressing low levels of COX-2. Apigenin suppresses the expression of COX-2 at the transcriptional and post-transcriptional levels [5,9]; thus, apigenin might have rendered the activated lupus cells dull-positive for COX-2 staining as well.

### Apigenin induces apoptosis of lupus immune cells

Apigenin is known to induce apoptosis of cancer cells [24,25], and it potentiates AICD in normal human T cells that are recurrently activated [5], which would guard against autoreactivity. We therefore examined the ability of apigenin to induce apoptosis of lupus immune cells, which are spontaneously activated *in vivo *from ongoing autoimmune response. Treatment with apigenin *in vitro *at 30 μM induced significant apoptosis of T cells, B cells, DCs, and macrophages of SNF1 mice after 24 hours of incubation as compared with cultures with vehicle (Figure 6d, *P < 0.001, **P < 0.01). At a concentration of 30 μM, apigenin induced twofold more apoptosis in DCs and macrophages than in T and B cells. At a concentration of 10 μM, apigenin did not induce significant apoptosis of T cells, but B cells, DCs, and macrophages were affected.

### Apigenin suppressed interleukin-6 production induced through Toll-like receptor-7 and -9 pathways

**Figure 7:**
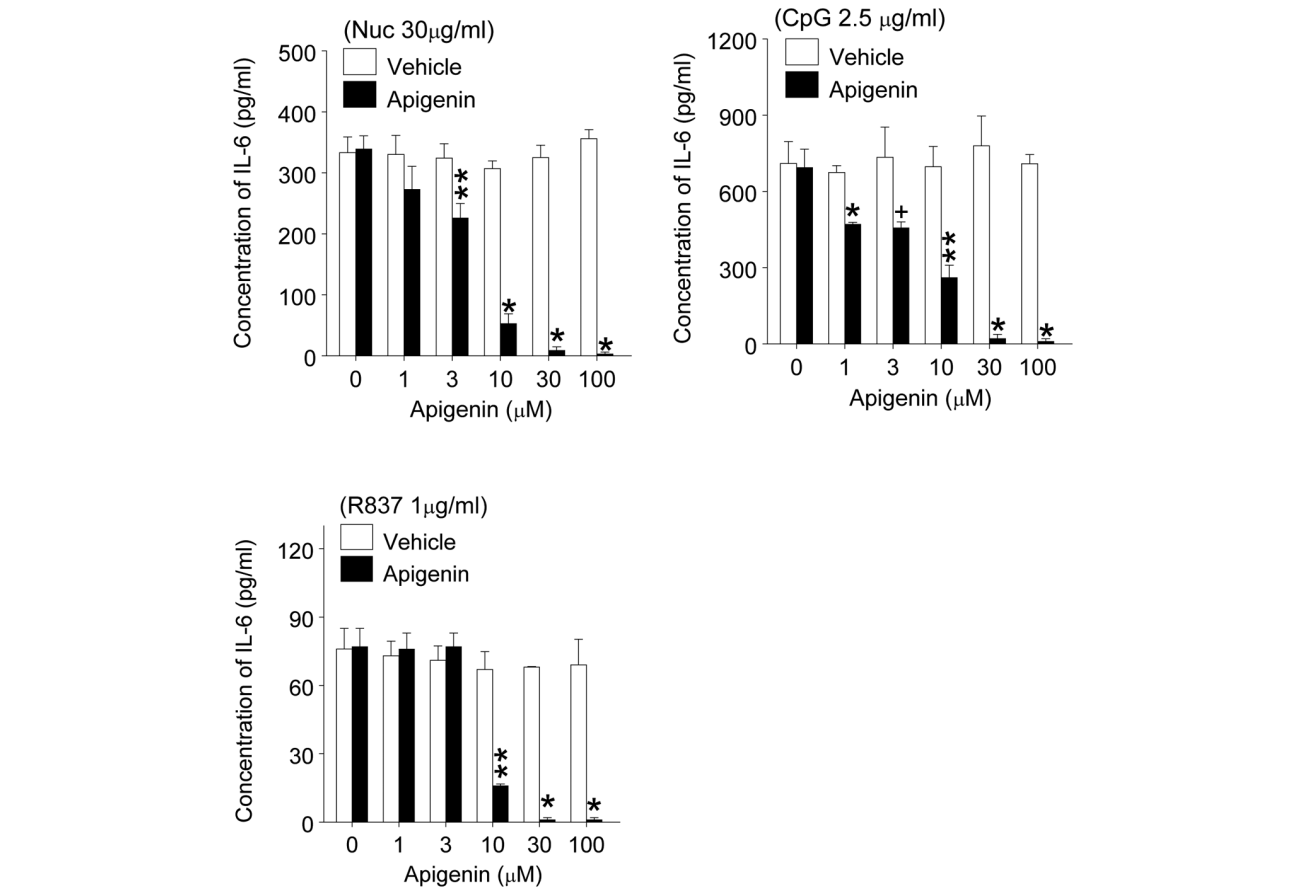
Apigenin markedly decreased interleukin-6 (IL-6) production by lupus dendritic cells (DCs). DCs from unmanipulated 3-month-old SNF1 mice were stimulated with nucleosomes (30 μg/mL), CpG (2.5 μg/mL), and R837 (1 μg/mL) in the presence of various amounts of apigenin. IL-6 in culture supernatant was measured by enzyme-linked immunosorbent assay. *P < 0.001, **P < 0.01, and ^+^*P *< 0.05. Nuc, nucleosome; SNF1, (SWR × NZB)F1.

IL-6 produced by APCs is important for generating Th17 cells [26], and apigenin suppressed Th17 responses in SNF1 mice (Figure 3a right panel and Figure 5b). Moreover, DNA and RNA in the major lupus autoantigens, nucleosomes and ribonucleoprotein (RNP), can act as TLR-9 and TLR-7 ligands, respectively [27]. We therefore tested whether apigenin could suppress IL-6 production stimulated by nucleosomes, CpG (TLR-9 ligand), and R837 (TLR-7 ligand) in SNF1 mice. Apigenin at a concentration of 30 μM suppressed IL-6 production induced by nucleosome, CpG, and R837 completely (Figure 7, *P *< 0.01 to 0.001), but significant inhibition was seen even at 1 μM (for response to CpG) and 3 μM (for nucleosome). Apigenin at concentrations of 30 to 100 μM also suppressed IFN-α production by DCs stimulated with 2.5 μg/mL CpG (*P *< 0.001), but not at concentrations of 1 to 10 μM (data not shown).

### Apigenin did not increase suppressive function of CD4^+^CD25^+ ^regulatory T cells

Since IL-6 inhibits regulatory T (T_reg_) cells while promoting Th17 cell expansion and we observed that apigenin suppressed IL-6 production by APCs, we analyzed whether apigenin could increase CD4^+^CD25^+^T_reg _cell activity. After 2 months of treatment, CD4^+^CD25^+^T cells from apigenin- or vehicle-treated SNF1 mice were cocultured with splenocytes from 4.5-month-old unmanipulated SNF1 mice in an autoantigen-specific suppression assay described previously [18,28]. As compared with CD4^+^CD25^+ ^T_reg _cells from vehicle-treated SNF1 mice, apigenin treatment did not increase the suppressive function of CD4^+^CD25^+ ^T cells on nucleosome-specific Th1 and Th17 responses (*P *< 0.05, data not shown).

## Discussion

Using SNF1 mice that spontaneously develop human lupus-like disease, we show that apigenin treatment *in vitro *and *in vivo *markedly inhibited autoimmune responses of Th1 and Th17 cells that are spontaneously primed to nucleosomes, the major nuclear autoantigen in lupus. Both IFN-γ-producing Th1 cells and IL-17-producing Th17 cells are critical for help in the production of pathogenic autoantibodies [17,22,29,30] and development of lupus nephritis [18,31-34]. Moreover, the spontaneously pre-primed, autoimmune Th17 cells in SNF1 mice with lupus-like disease can expand when challenged with nucleosomes *ex vivo *without requiring any polarizing cytokine conditions or PMA (phorbol myristate acetate)-ionomycin additions that are used widely to detect such pathogenic Th cells in other systems [18]. Apigenin suppressed production of the Th17-inducing cytokine, IL-6, by APCs stimulated by nucleosomes, CpG (TLR-9 ligand), and R837 (TLR-7 ligand). This is relevant because DNA and RNA in the major lupus autoantigens, nucleosomes and RNP, can stimulate APCs via TLR-9 and TLR-7 pathways, respectively [27]. Consequent to the inhibition of lupus Th cells, apigenin treatment suppressed the production of IgG class-switched pathogenic autoantibodies to nuclear antigens and significantly delayed the development of severe glomerulonephritis (Figures 1, 2, 3 and 4).

However, autoantigen-presenting function of APCs appeared to be more sensitive to the inhibitory effect of apigenin, although apigenin has been shown to inhibit NF-κB activation pathways in both T cells [5] and macrophages [35,36]. Macrophages and myeloid DCs are important for ongoing presentation of nucleosome-derived epitopes to autoreactive T cells in mice with established lupus [37,38], and hyperactive APCs are a characteristic feature of lupus, playing a critical role in initiation and pathogenesis [39-43]. By inhibiting NF-kB activation, not only does apigenin inhibit the autoantigen-presenting and stimulatory functions of the APCs necessary for activation and expansion of autoreactive Th and B cells, but it causes apoptosis of the hyperactive lupus APCs (this study), probably by inhibiting NF-kB-regulated anti-apoptotic molecules, especially COX-2 and c-FLIP [5,6]. However, the functional inhibitory effect of apigenin in vitro could be seen in concentrations of as low as 0.3 to 3 mM (Figures 1a and 7), which were well below the concentrations (10 to 30 mM) required for inducing significant apoptosis (Figure 6c).

Despite the fact that apigenin is widely distributed in fruits and herbs, diet is insufficient for bioavailable therapeutic levels of apigenin due to first-pass metabolism (glucuronidation) in gut and liver, although some systemic effects of diets rich in apigenin are detectable [44]. Bioavailability has been improved in the case of other drugs by the pharmaceutical industry, and similar attempts are being applied to related flavone compounds [45]. Thus, apigenin, a non-mutagenic plant flavone, is a strong inhibitor of NF-κB activation and COX-2 expression in activated autoimmune cells, but it also has properties that might reduce the risk of coronary disease, as mentioned above. Obviously, relatively benign COX-2 and NF-κB inhibitors such as apigenin and other herbal products [46] might be of value in lupus therapy.

## Conclusions

Apigenin inhibits autoantigen-presenting and stimulatory functions of the APCs necessary for activation and expansion of autoreactive Th1 and Th17 cells and B cells in lupus. Apigenin also causes apoptosis of the hyperactive lupus APCs, T cells, and B cells, probably by inhibiting expression of NF-κB-regulated anti-apoptotic molecules, especially COX-2 and c-FLIP, which are persistently hyperexpressed by the lupus immune cells. Although apigenin, a non-mutagenic plant flavone, inhibits COX-2 expression in activated autoimmune cells, it also has properties that might reduce the risk of coronary disease in contrast to conventional COX-2 inhibitors. Increasing the bioavailability of simple dietary plant-derived COX-2 and NF-κB inhibitors, such as apigenin, might be of value in lupus therapy as well as for suppressing inflammation in other Th17-mediated inflammatory diseases like rheumatoid arthritis, Crohn disease, and psoriasis and in prevention of inflammation-based tumors overexpressing COX-2 (colon, breast).

## Abbreviations

ACUC: Animal Care and Use Committee; AICD: activation-induced cell death; AP: alkaline phosphatase; APC: antigen-presenting cell; c-FLIP: cellular FLICE-like inhibitory protein; COX-2: cyclooxygenase 2; DC: dendritic cell; DMSO: dimethyl sulfoxide; dsDNA: double-stranded DNA; ELISA: enzyme-linked immunosorbent assay; ELISPOT: enzyme-linked immunosorbent spot; IFN-γ: interferon-gamma; IL: interleukin; NF-κB: nuclear factor-kappa-B; PBS: phosphate-buffered saline; RNP: ribonucleoprotein; SNF1: (SWR × NZB)F1; ssDNA: single-stranded DNA; Th: T helper; TLR: Toll-like receptor; T_reg_: regulatory T.

## Competing interests

The authors declare that they have no competing interests.

## Authors' contributions

H-KK participated in study design, apigenin therapy, cellular immunologic assays, acquisition of data, statistical analysis, and drafting of the manuscript. DE measured levels of autoantibodies and assisted in apigenin therapy injections, cellular immunologic assays, and data acquisition. ML assisted in cellular immunologic assays. SKD conceived of the study and participated in its design and coordination, data analysis, and manuscript preparation. All authors read and approved the final manuscript.
